# Using Cu as a Spacer to Fabricate and Control the Porosity of Titanium Zirconium Based Bulk Metallic Glass Foams for Orthopedic Implant Applications

**DOI:** 10.3390/ma15051887

**Published:** 2022-03-03

**Authors:** Pei-Chun Wong, Sin-Mao Song, Pei-Hua Tsai, Muhammad Jauharul Maqnun, Wei-Ru Wang, Jia-Lin Wu, Shian-Ching (Jason) Jang

**Affiliations:** 1 Department of Orthopedics, School of Medicine, College of Medicine, Taipei Medical University, Taipei 110301, Taiwan; pcwong0424@tmu.edu.tw; 2 Department of Orthopedics, Taipei Medical University Hospital, Taipei 110301, Taiwan; 3 Orthopedics Research Center, Taipei Medical University Hospital, Taipei 110301, Taiwan; 4 Institute of Materials Science and Engineering, National Central University, Taoyuan 320317, Taiwan; bear82112760103@gmail.com (S.-M.S.); peggyphtsai@gmail.com (P.-H.T.); joeharley7@gmail.com (M.J.M.); 5 School of Biomedical Engineering, College of Biomedical Engineering, Taipei Medical University, Taipei 110301, Taiwan; amber16743@tmu.edu.tw; 6 Centers for Regional Anesthesia and Pain Medicine, Wan Fang Hospital, Taipei Medical University, Taipei 110301, Taiwan; 7 Department of Mechanical Engineering, National Central University, Taoyuan 320317, Taiwan

**Keywords:** porous, TiZr-based bulk metallic glass, Cu spacer, biocompatibility, mechanical properties controllability

## Abstract

In this study, a porous titanium zirconium (TiZr)-based bulk metallic foam was successfully fabricated using the Cu spacer by employing the hot press method. TiZr-based bulk metallic foams with porosities ranging from 0% to 50% were fabricated and analyzed. The results indicate that thermal conductivity increased with the addition of Cu spacer; the increased thermal conductivity reduced the holding time in the hot press method. Moreover, the compressive strength decreased from 1261 to 76 MPa when the porosity of the TiZr-based bulk metallic foam increased to 50%, and the compressive strength was predictable. In addition, the foam demonstrated favorable biocompatibility in cell viability, cell migration capacity, and calcium deposition tests. Moreover, the pore size of the porous TiZr-based bulk metallic foam was around 120 µm. In conclusion, TiZr-based bulk metallic foam has favorable biocompatibility, mechanical property controllability, and porous structure for bone ingrowth and subsequent enhanced osteointegration. This porous TiZr-based bulk metallic foam has great potential as an orthopedic implant to enhance bone healing and decrease healing time.

## 1. Introduction

Metallic materials are usually used in orthopedic implants such as bone screws and plates, artificial joints, and spinal fusion devices. To achieve the biological fixation of orthopedic implants with the bone, surface coating, surface modification, and bone cement have been employed in the manufacturing of orthopedic implants to improve bone–implant healing [1,2,3]. However, inadequate binding strength is noted at the contact area between an orthopedic implant and the bone tissue. The ingrowth of osteoblasts into an implant could improve the integration of the implant into the bone [4]. Porous metallic materials have been used as an orthopedic scaffold to improve biological fixation because bone ingrowth can occur around the porous surface in the porous metallic scaffold [5]. 

An ideal porous metallic material should have the following characteristics: (1) satisfactory biocompatibility, (2) osteoconductive and osteoinductive abilities for improving bone healing, (3) adequate mechanical properties for structural support and load bearing, (4) suitable pore size for cell and tissue ingrowth, and (5) open-cell structure for allowing cell and tissue ingrowth [6,7,8]. However, the recently developed porous metallic material as a scaffold still has some drawbacks. First, the absence of biocompatibility and corrosion or wear in the body may release toxic ions or metallic particles, leading to inflammation and subsequent surrounding tissue damage. Second, the Young’s modulus of metals is usually higher than that of the bone tissue, thus exerting a stress-shielding effect [5,9].

Titanium zirconium (TiZr)-based bulk metallic glass (BMG) has recently attracted attention for its suitability in biomedical applications because of its more favorable biocompatibility, corrosion behavior, and mechanical properties compared with those of TiZr-based metallic materials. TiZr-based BMG has demonstrated excellent biocompatibility not only in in vitro studies but also in in vivo trials with osteoinduction and without local inflammation [10,11,12,13,14]. 

Porous materials should have a pore size and an open-pore structure similar to the bone for their use as an orthopedic implant material. The capability of implant materials to cause new bone regeneration and form strong fixation with the applied tissue depends on the interconnected bonding of porous materials [15]. The design of a porous material can reduce mismatch issues in mechanical properties, especially Young’s modulus. Reducing implant stiffness might prevent the stress-shielding effect, which causes implant loosening and bone resorption [16,17]. Porous material in metallic and BMG foams can be classified as having open-cell and closed-cell porosity. A porous material with closed-cell porosity is defined as that being fully closed by a thin wall without interconnecting with other cells. Metallic foams with closed-cell porosity are usually developed using a random foaming process in which the size, shape, and location of pores differ depending on fabrication parameters. In a porous material with open-cell porosity, individual cells are interconnected with each other, allowing the tissue to infiltrate the foam.

Porous materials can be manufactured by many techniques for biomedical use, such as additive manufacturing (AM) technology, selective laser sintering (SLS), and selective laser melting (SLM) [18]. Additive manufacturing is also named 3D printing; this method produces the product layer by layer. At present, there are two categories of AM technologies suitable for metallic porous materials: powder bed fusion technology (PBF) and directional deposition technology (DED) [19]. SLS and SLM are basically the same techniques, using a laser as an energy source to sinter or melt the metallic powders, but the laser energy of SLM is much higher than that of SLM [20,21]. However, the manufacturing of metallic glass powders by SLM is difficult for now [22]; moreover, the low glass-forming ability and poor thermal stability of most metallic glass systems may lead to the crystallization problem [23].

For the fabrication of porous TiZr-based BMG foams, adding spacer into the matrix powders for hot pressing is an easy way of fabricating the foam material. Nguyen et al. have successfully fabricated TiZr-based BMG foams by using different volume percentages of NaCl and Al spacers [13,14]. In this study, we used Cu spacer to fabricate porous Ti_42_Zr_35_Ta_3_Si_5_Co_12.5_Sn_2.5_ BMG foam by using the hot press method. We examined and compared the porosity, thermal conductivity, glass-forming ability, microstructure, mechanical properties, cell viability, and biological responses of Ti_42_Zr_35_Ta_3_Si_5_Co_12.5_Sn_2.5_ BMG foams with different porosities. We hypothesized that mechanical properties can be controlled and that mechanical properties would decrease with increased porosity. Moreover, we hypothesized that no significant differences would be observed between material properties and cell responses. The aim of this study was to fabricate porous TiZr-based BMG foams with suitable mechanical strength and good biocompatibility for orthopedic implant applications.

## 2. Materials and Methods

### 2.1. Study Design

In this study, we used different volume fractions of Cu particles as a spacer to fabricate Ti_42_Zr_35_Ta_3_Si_5_Co_12.5_Sn_2.5_ BMG foams with different porosities by adopting the hot press and immersion methods. We examined and compared the thermal conductivity, glass-forming ability, density, porosity, microstructure, surface morphology, mechanical properties, corrosion behavior, and biocompatibility of BMG foams fabricated using different volume fractions of Cu particles. The volume fractions of Cu particles were set as 0, 10, 20, 30, 40, and 50 vol.%.

### 2.2. Sample Preparation

Ti_42_Zr_35_Ta_3_Si_5_Co_12.5_Sn_2.5_ powders were fabricated using the atomization process under argon atmosphere and sorted based on particle size. Subsequently, 25 μm sized Ti_42_Zr_35_Ta_3_Si_5_Co_12.5_Sn_2.5_ powders were mixed with 120 µm sized Cu spacer particles to obtain particles of different porosities (0%, 10%, 20%, 30%, 40%, and 50%). The well-mixed particles were used in the hot press method in which a hot press pressure of 300 MPa and a hot press temperature of 520 °C were applied for a holding time of 5 min to prepare BMG foam. Subsequently, the samples were polished, and the foam was placed into the mixed solution of HNO_3_ and H_2_O at a concentration of 1:1 to remove the Cu spacer.

### 2.3. Real Porosity Test and Density Test

The real porosity of Ti_42_Zr_35_Ta_3_Si_5_Co_12.5_Sn_2.5_ BMG foam was analyzed using the Archimedes method. We first measured the volume of Ti_42_Zr_35_Ta_3_Si_5_Co_12.5_Sn_2.5_ BMG foam in which the spacer was not removed and calculated the mass deviation of the sample in air and in water. Second, we immersed Ti_42_Zr_35_Ta_3_Si_5_Co_12.5_Sn_2.5_ BMG foam in which the spacer was removed in water again to measure the volume of pores and voids created by the removal of spacers by determining the amount of water penetrating into the foam. The real porosity was calculated as the ratio of the original volume to the volume of pores and voids using the following Equations (1)–(3):

(1) $$V_{1}=\frac{m_{air}-m_{water}}{D}$$

(2) $$V_{2}=\frac{m_{air}-m_{water}}{D}$$

(3) $$Real\;porosity\;\left(\%\right)=\frac{V_{2}}{V_{1}}\times 100$$

where $V_{1}$ and $V_{2}$ are the original volume of the sample and the volume of pores and voids, respectively. $m_{air}$ and $m_{water}$ are the weights of the sample determined by placing it in air and water before the removal of the spacer, respectively. $m_{air}$ and $m_{water}$ are the weights of the sample determined by placing it in the air and water after the removal of the spacer. D is the density of water.

The density of Ti_42_Zr_35_Ta_3_Si_5_Co_12.5_Sn_2.5_ BMG foams was measured using the Archimedes method [24]. The samples were weighed both in air and water, and their density was calculated using the following Equation (4):

(4) $$\rho =\frac{m_{air}}{m_{air}-m_{water}}+D$$

where ρ is the density of the sample; $m_{air}$ and $m_{water}$ are the weights of the sample in air and water, respectively; and *D* is the density of water.

### 2.4. Thermal Conductivity Test and Glass-Forming Ability Analysis

Thermal conductivity is related to the efficiency of holding time during the hot press. In other words, a higher thermal conductivity of samples in the hot press may produce higher mechanical properties of samples. Ti_42_Zr_35_Ta_3_Si_5_Co_12.5_Sn_2.5_ BMG foams with Cu spacers were fabricated using a cylinder with a diameter of 7 mm and a thickness of 2 mm by using the hot press machine. Their thermal conductivity was measured using the thermal property tester (LFA 467 HT HyperFlash, Netzsch, Selb, Germany). To calculate thermal conductivity, the density and specific heat capacity were measured using the Archimedes method and a differential scanning calorimeter (DSC), respectively. Finally, the thermal conductivity of the samples was calculated using the following Equation (5):

(5) k=α×ρ×∁ρ

where k is the thermal conductivity (W/mK), α is the thermal diffusivity (mm^2^/s), ρ is the density (g/cm^2^), and ∁ρ is the specific heat capacity of the samples (J/gK).

The glass-forming ability of Ti_42_Zr_35_Ta_3_Si_5_Co_12.5_Sn_2.5_ foams without Cu spacers was analyzed using a DSC (DSC404, Netzsch, Selb, Germany) at a heating rate of 40 K/min.

### 2.5. Microstructure Analysis

The microstructure of Ti_42_Zr_35_Ta_3_Si_5_Co_12.5_Sn_2.5_ BMG foams was examined through X-ray diffraction (XRD) analysis by using the Bruker D8A X-ray diffractometer (Bruker, Bremen, Germany) with monochromatic Cu K𝛼 radiation. In addition, Ti_42_Zr_35_Ta_3_Si_5_Co_12.5_Sn_2.5_ BMG foams with 10% porosity were analyzed using a transmission electron microscope (TEM; JEOL JEM2100, Tokyo, Japan). The thin-foil specimen required for TEM analysis was prepared using a focused ion beam system (Versa 3D Dual Beam, FEI, Hillsboro, OR, USA).

### 2.6. Morphology Observation

The surface morphology of Ti_42_Zr_35_Ta_3_Si_5_Co_12.5_Sn_2.5_ BMG foams was examined using a scanning electron microscope (SEM; Inspect F50, FEI, Hillsboro, OR, USA) to determine the pore size and distribution of BMG foams.

### 2.7. Mechanical Property Test and Prediction

We performed the uniaxial compression test to determine the compressive strength and Young’s modulus of Ti_42_Zr_35_Ta_3_Si_5_Co_12.5_Sn_2.5_ BMG foams. The test samples were first cut into a size of 2.5 mm × 2.5 mm × 5 mm. A universal mechanical test system (Hung Ta, HT9102, Taipei, Taiwan) was used to perform a compression test at a strain rate of 1 × 10^−4^ mm/s.

The Gibson and Ashby model was used to predict the Young’s modulus and compressive strength of Ti_42_Zr_35_Ta_3_Si_5_Co_12.5_Sn_2.5_ BMG foams based on their relationship with relative density. According to the model, the desired mechanical properties can be estimated and adjusted by controlling the real porosity of samples. The relationships between Young’s modulus and relative density and between compressive strength and relative density were calculated using the following Equations (6) and (7):

(6) $$\frac{E}{E_{s}}=C1{\left(\frac{\rho }{\rho_{s}}\right)}^{n1}$$

(7) $$\frac{\sigma }{\sigma_{s}}=C2{\left(\frac{\rho }{\rho_{s}}\right)}^{n2}$$

where E, σ, and ρ are the elastic modulus, compressive strength, and density of a porous sample, respectively. $E_{s}$, $\sigma_{s}$, and $\rho_{s}$ are the elastic modulus, compressive strength, and density of the open-cell wall material, respectively. C1, C2, n1, and n2 are dependent on the bonding-force interface between amorphous metallic glass particles and the structure of porous samples. Our previous study reported that the $E_{s}$, $\sigma_{s}$, and $\rho_{s}$ of Ti_42_Zr_35_Ta_3_Si_5_Co_12.5_Sn_2.5_ BMG foams were approximately 112 GPa, 1342 MPa, and 6.08 g/cm^3^, respectively [13].

### 2.8. Biocompatibility Test

To prepare the precipitation medium, Ti_42_Zr_35_Ta_3_Si_5_Co_12.5_Sn_2.5_ BMG foams with a dimension of 2.5 mm × 8 mm × 8 mm and different porosities were first sterilized and immersed in 100 mL of alpha-minimum essential medium (Gibco, NY, USA) containing 10% fetal bovine serum. After 14 days, the samples immersed in the culture medium were removed and the precipitation medium was stored at 4 °C in a refrigerator for further use.

The biocompatibility of Ti_42_Zr_35_Ta_3_Si_5_Co_12.5_Sn_2.5_ BMG foams was examined using the 3-(4,5-dimethylthiazol-2-yl)-2,5-diphenyltetrazolium bromide (MTT) assay involving indirect contact with MC3T3-E1 preosteoblasts. We dispensed 100 μL of MC3T3-E1 preosteoblast suspension into a 96-well plate to achieve a cell density of 3000 cells/well and preincubated the plate in the incubator at 37 °C under 5% CO_2_ atmosphere for cell attachment. After 24 h, we added 10 μL of the precipitation medium into each well and incubated the plate for another 24 h in the incubator. Next, 10 μL of MTT solution (Invitrogen) was gently added to each well, and the cells were cultured for 3 h. Subsequently, 100 μL of dimethylsulfoxide was added to the wells, and the solution was mixed well. Finally, optical density was measured using an enzyme-linked immunosorbent assay reader (Multiskan FC; Thermo, Waltham, MA, USA) at a wavelength of 560 nm.

The migration capacity of MC3T3-E1 preosteoblasts was determined using the scratch assay with the simulation of the precipitation medium. The MC3T3-E1 preosteoblast suspension was seeded into a 24-well culture plate with a cell density of 5000 cells/well and incubated for 24 h. A straight line was scratched using a 1000 μL pipette tip along the monolayer of cells. The culture medium was used to gently remove the cell debris. Subsequently, 500 μL of the precipitation medium was added to each well, followed by 8 h of incubation. After the incubation, the cells were observed under an optical microscope (Primovert; Zeiss, Jena, Germany) and images were analyzed using Image J software.

Extracellular calcium deposition in MC3T3-E1 preosteoblasts was analyzed by performing alizarin red S (ARS) staining. First, 500 μL of the MC3T3-E1 preosteoblast suspension was seeded into a 24-well culture plate with a cell density of 5000 cells/well. After 24 h incubation for cell attachment, the standard culture medium was replaced with the precipitation medium; the plate was then incubated for 21 days. The precipitation medium was changed every 3 days. Before staining, the precipitation medium was discarded, and the cells were gently rinsed with phosphate-buffered saline three times. Subsequently, the cells were fixed with 4% paraformaldehyde for 15 min at room temperature. The fixative was removed, and the cells were stained with ARS dye for 20 min at room temperature in dark. After the removal of the ARS dye, the cells were observed under an optical microscope (Primovert, Zeiss, Jena, Germany). The stained area was detected and quantified using Image J software (version 1.53K, National Institutes of Health, Bethesda, MD, USA).

### 2.9. Statistical Analysis

The biocompatibility results are presented as the mean ± standard deviation. All statistical analyses were performed using SPSS version 20. One-way analysis of variance with post hoc Tukey’s tests and independent-sample t tests were performed to analyze data. Statistical significance was considered if *p* < 0.05.

## 3. Results

### 3.1. Real Porosity

Table 1 lists the real porosity values. Ti_42_Zr_35_Ta_3_Si_5_Co_12.5_Sn_2.5_ BMG foams with a real porosity of 2.0%, 11.1%, 27.6%, 40.4%, 51.0%, and 67.9% were fabricated using the hot press method with the volume fractions of 0%, 10%, 20%, 30%, 40%, and 50% of Cu spacer particles. 

### 3.2. Thermal Conductivity

**Figure 1:**
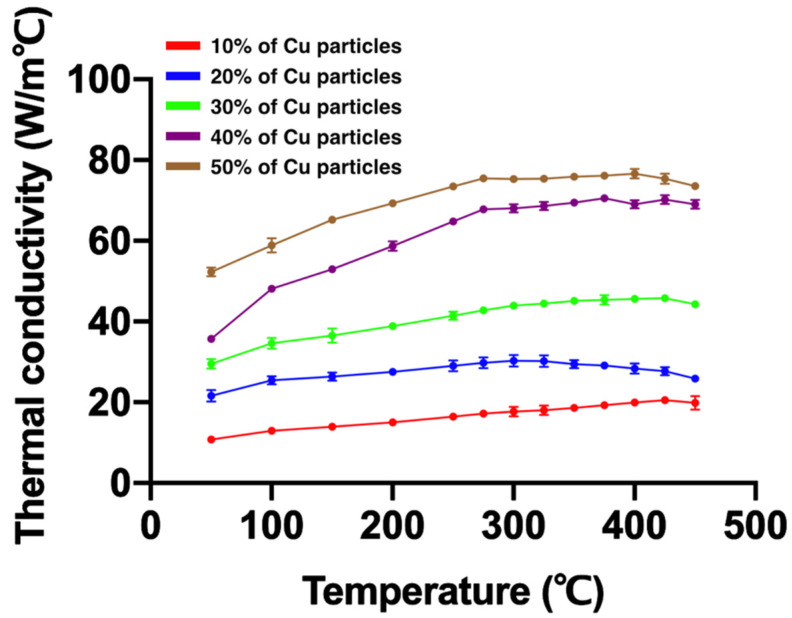
Thermal conductivity of Ti_42_Zr_35_Ta_3_Si_5_Co_12.5_Sn_2.5_ bulk metallic glass foams fabricated using different volume fractions of Cu spacer particles. A higher volume fraction of Cu particles resulted in higher thermal conductivity.

Figure 1 and Table 2 present the thermal conductivity of Ti_42_Zr_35_Ta_3_Si_5_Co_12.5_Sn_2.5_ BMG foams fabricated using different volume fractions of Cu spacer particles. As shown in Figure 1, Cu spacer particles with a higher volume fraction resulted in higher thermal conductivity. The thermal conductivity of all samples increased with the increase in temperature.

### 3.3. Glass-Forming Ability

Table 3 lists the glass-forming abilities of Ti_42_Zr_35_Ta_3_Si_5_Co_12.5_Sn_2.5_ BMG foams with different porosities. The foam samples fabricated with and without Cu spacer particles exhibited a high glass-forming ability with a similar glass transition temperature ($T_{g}$) and crystallization temperature ($T_{x}$), as well as demonstrating a large supercooled liquid region. The glass-forming abilities of Ti_42_Zr_35_Ta_3_Si_5_Co_12.5_Sn_2.5_ BMG foams with different porosities fabricated using Cu spacer particles were similar. The porous structure did not affect the glass-forming ability.

### 3.4. Microstructure Analysis

**Figure 2:**
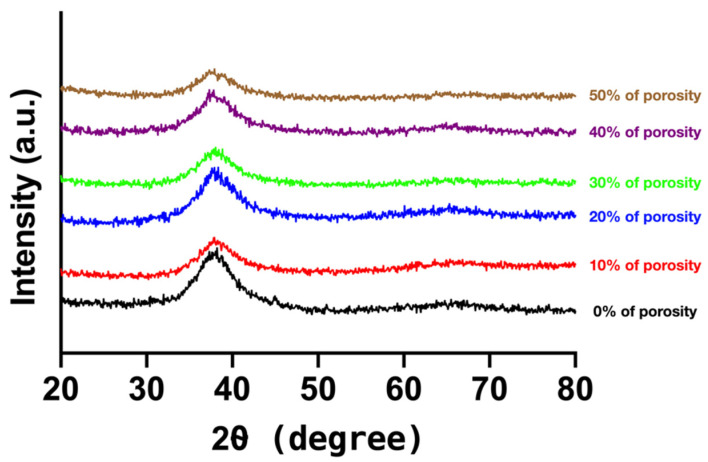
X-ray diffraction pattern of Ti_42_Zr_35_Ta_3_Si_5_Co_12.5_Sn_2.5_ bulk metallic glass foams fabricated using Cu spacer particles by employing the hot press method.

Figure 2 depicts the XRD pattern of Ti_42_Zr_35_Ta_3_Si_5_Co_12.5_Sn_2.5_ BMG foams. The XRD patterns of all samples exhibited a typically broad diffraction peak at 30–50°, indicating that porous Ti_42_Zr_35_Ta_3_Si_5_Co_12.5_Sn_2.5_ BMG foams fabricated using the hot press method with different volume fractions of Cu spacer particles remained in the amorphous state. No crystalline peak was observed.

**Figure 3:**
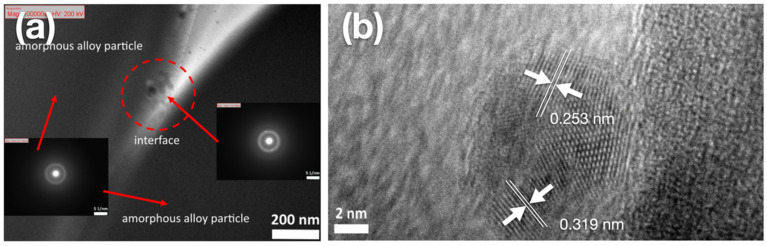
High-resolution transmission electron microscope images of Ti_42_Zr_35_Ta_3_Si_5_Co_12.5_Sn_2.5_ bulk metallic glass foams fabricated using 10 vol.% of Cu spacer particles. (**a**) Selected area electron diffraction (SAED) pattern and (**b**) nanocrystallization zone.

Figure 3 shows the TEM finding of the interface of bonding between the amorphous alloy particles of Ti_42_Zr_35_Ta_3_Si_5_Co_12.5_Sn_2.5_ BMG foams fabricated using 10 vol.% of Cu spacer particles. Figure 3a presents a typical hollow ring by the selected area electron diffraction pattern. This finding indicated that the sample remained in the amorphous state during the hot press process and demonstrated a strong bonding-force interface between amorphous alloy particles. However, few lattices were observed in the amorphous phase, indicating that nanocrystallization did not occur in the observed sample based on the analysis of the selected parameters of hot pressing. As shown in Figure 3b, nanocrystalline phases contained the normal α-Ti phase (HCP structure) and β-Ti phase (BCC structure) with lattice constants of 0.253 and 0.319 nm, respectively. Thus, the nanocrystalline phases observed at the interfaces of TiZr-based BMG foams fabricated using Cu spacers can be attributable to Cu particles promoting the formation of normal α-Ti and β-Ti phases.

### 3.5. Morphology Observation

**Figure 4:**
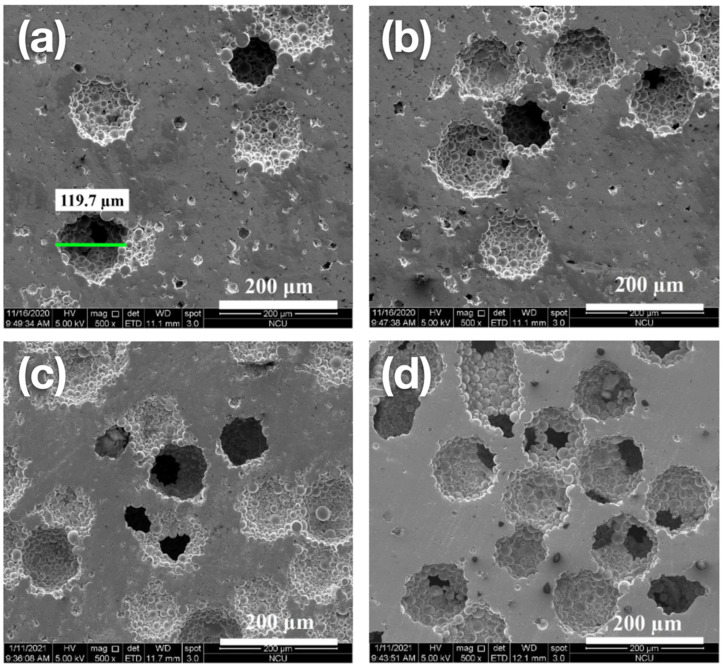
Scanning electron microscope images depicting the surface morphology of Ti_42_Zr_35_Ta_3_Si_5_Co_12.5_Sn_2.5_ bulk metallic glass foams with porosities of (**a**) 10%, (**b**) 20%, (**c**) 30%, and (**d**) 40%. These images indicate the open-cell structure of foams and that the pore size was approximately 120 µm.

Figure 4 shows the SEM findings of the surface morphology of Ti_42_Zr_35_Ta_3_Si_5_Co_12.5_Sn_2.5_ BMG foams with different porosities fabricated using Cu spacer particles by employing the hot press method. The Ti_42_Zr_35_Ta_3_Si_5_Co_12.5_Sn_2.5_ powder could be clearly observed, and the binding interaction between each powder remained strong. The pore dimension in Figure 4a was 119.7 µm, which was matched with the Cu spacer particles.

### 3.6. Mechanical Properties and Their Prediction

**Figure 5:**
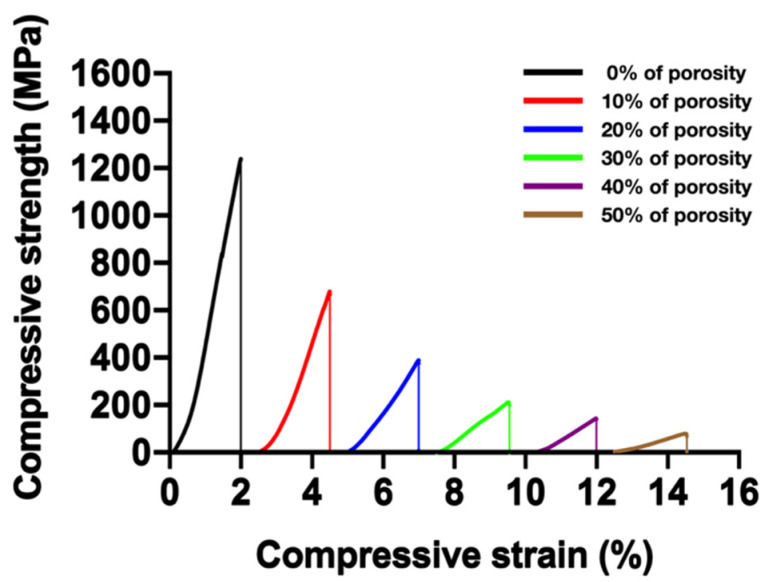
Stress–strain curve of Ti_42_Zr_35_Ta_3_Si_5_Co_12.5_Sn_2.5_ bulk metallic glass foams with different porosities. The compressive strength decreased with increasing porosity.

The mechanical properties of Ti_42_Zr_35_Ta_3_Si_5_Co_12.5_Sn_2.5_ BMG foams were examined using the compression test. The foams with porosities of 0%, 10%, 20%, 30%, 40%, and 50% presented compressive strengths of 1261, 665, 388, 214, 144, and 76 MPa, respectively (Figure 5). Table 4 lists the mechanical properties of Ti_42_Zr_35_Ta_3_Si_5_Co_12.5_Sn_2.5_ BMG foams. Young’s modulus decreased from 79.7 to 4.6 GPa for the foam with a porosity of 50%. The results indicated that mechanical strength decreased with increased porosity. In addition, the mechanical properties could be controlled by adjusting the porosity.

**Figure 6:**
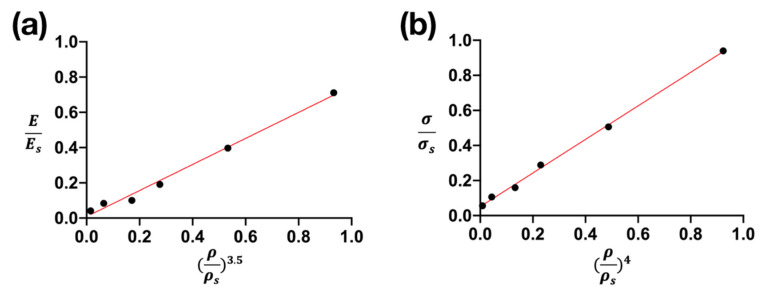
Prediction of (**a**) Young’s modulus and (**b**) compressive strength of Ti_42_Zr_35_Ta_3_Si_5_Co_12.5_Sn_2.5_ bulk metallic glass foams by using the Gibson and Ashby model.

Figure 6 shows the linear fitting of Young’s modulus and compressive strength with a relative density of Ti_42_Zr_35_Ta_3_Si_5_Co_12.5_Sn_2.5_ BMG foams determined using the Gibson and Ashby model. The relationship between the Young’s modulus and relative density of BMG foams is expressed as E/Es = 0.752 (ρ/ρs)3.5 and R2 = 0.995. The relationship between compressive strength and relative density is expressed as σ/σs = 1.04 (ρ/ρs)4 and R2 = 0.991. The aforementioned results indicated that the dependency of the Young’s modulus and compressive strength of TiZr-based BMG foams on relative density could be accurately predicted using the Gibson and Ashby model.

### 3.7. Cell Viability

**Figure 7:**
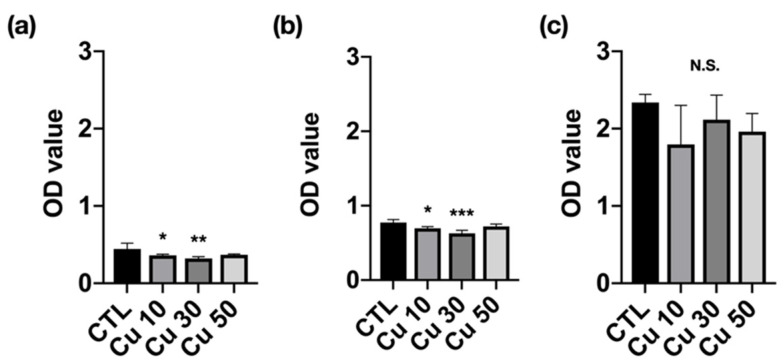
Cell viability of MC3T3-E1 preosteoblasts cultured with the precipitation medium of Ti_42_Zr_35_Ta_3_Si_5_Co_12.5_Sn_2.5_ bulk metallic glass foams with porosities of 10%, 30%, and 50% fabricated using different volume fractions of Cu spacer particles and immersion for (**a**) 1-, (**b**) 3-, and (**c**) 7-day incubation (*N* = 5 per group; * *p* < 0.05, ** *p* < 0.01, and *** *p* < 0.005; N.S., not significant).

Figure 7 shows the viability of MC3T3-E1 preosteoblasts cultured in different precipitation media. The Ti_42_Zr_35_Ta_3_Si_5_Co_12.5_Sn_2.5_ BMG foams with porosities of 10% and 30% demonstrated significantly lower cell viability at 1- and 3-day incubation. However, the cell viability at 7-day incubation did not differ between the foams with porosities of 10% and 30% and the control group. The optical density of all foam samples increased with an increase in incubation time, indicating that the cells were continually growing. Moreover, all the groups were determined as having first-level cytotoxicity according to ISO 10993-5 [25].

### 3.8. Cell Migration Capacity

**Figure 8:**
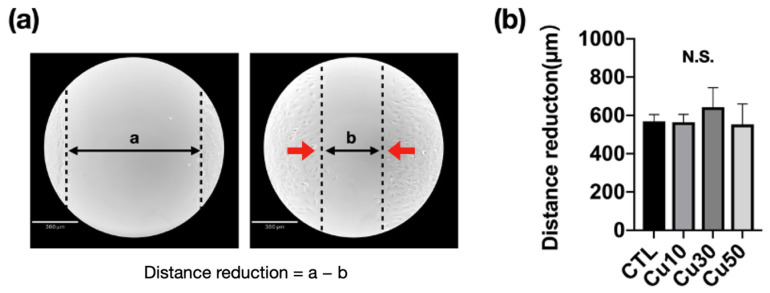
Cell migration capacity of MC3T3-E1 preosteoblasts cultured with the precipitation medium of Ti_42_Zr_35_Ta_3_Si_5_Co_12.5_Sn_2.5_ bulk metallic glass foams with porosities of 10%, 30%, and 50% fabricated using different volume fractions of Cu spacer particles. (**a**) Cell migration at the first scratch and after 8 h of incubation (control, CTL); (**b**) the distance of the gap decreased due to the migration of MC3T3-E1 preosteoblasts after 8 h of incubation (*N* = 5 per group; N.S., not significant).

Figure 8 shows the migration capacity of MC3T3-E1 preosteoblasts cultured with standard and different precipitation media after the scratch test and 8 h after incubation. The gap distance was measured using Image J software (version 1.53K, National Institutes of Health, Bethesda, MD, USA) to determine the migration of MC3T3-E1 preosteoblasts (Figure 8a). After 8 h of incubation, the gap distance decreased to approximately 600 μm. However, no significant difference was observed between the groups (Figure 8b).

### 3.9. Calcium Deposition

**Figure 9:**
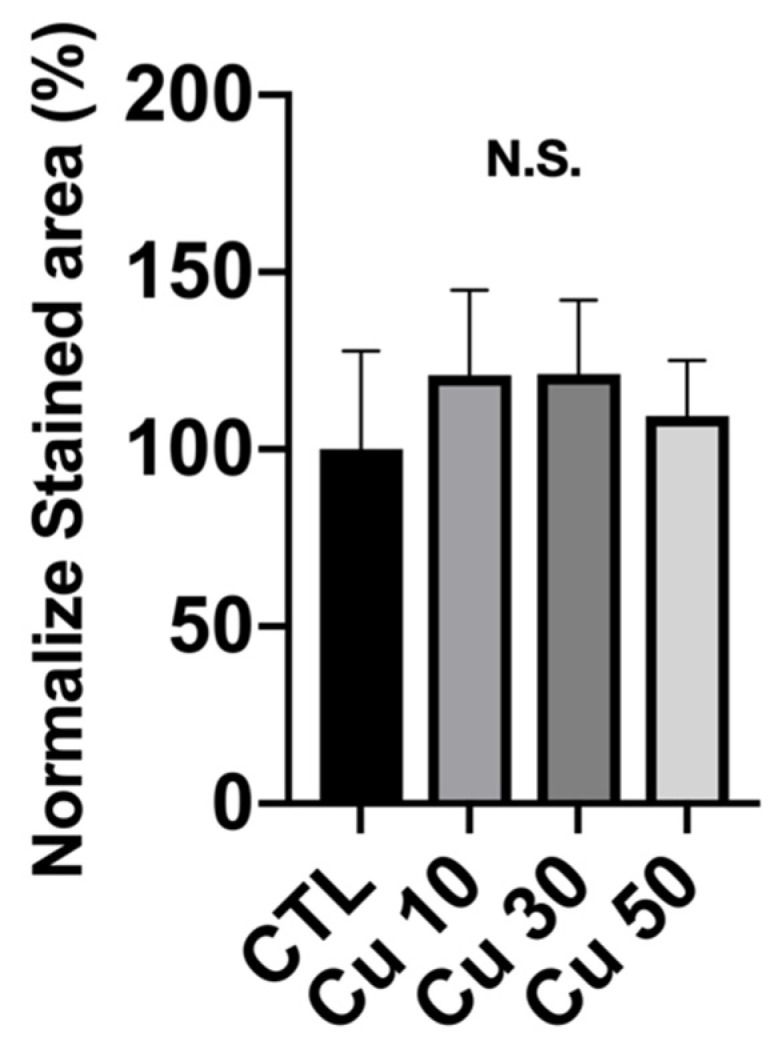
Extracellular matrix calcium and mineral deposition from MC3T3-E1 preosteoblasts cultured with the precipitation medium of Ti_42_Zr_35_Ta_3_Si_5_Co_12.5_Sn_2.5_ bulk metallic glass foams with porosities of 10%, 30%, and 50% fabricated using different volume fractions of Cu spacer particles and stained by alizarin red S dye. The results of the stained area of each experimental group are normalized to the control group (*N* = 5 per group; N.S., not significant).

To determine the applicability of a bone implant in orthopedic fields, calcium deposition is the first cell functional response that should be examined. Figure 9 shows the quantitative analysis of the area stained by ARS dye after normalization. The normalized calcium deposition rate in the precipitation medium of Ti_42_Zr_35_Ta_3_Si_5_Co_12.5_Sn_2.5_ BMG foams with porosities of 10%, 30%, and 50% was 120% ± 24%, 121% ± 20%, and 109% ± 15%, respectively (Figure 9). However, no significant difference in calcium deposition was observed between the groups.

## 4. Discussion

The hot press method is used to fabricate a porous metallic material. The parameters of the hot press process in sintering include the hot press temperature, holding time, and pressure. Under the same hot press temperature, holding time, and pressure, the high thermal conductivity of materials presented a stronger bonding interface between the particles and mechanical properties [13,14,26]. In other words, a material with a higher thermal conductivity would require a lower hot press temperature and holding time to fabricate a material with a uniform structure. For porous BMG foam fabrication, higher hot press temperature reaching the crystallization temperature and longer holding time may affect the hot surface of the material and cause crystallization in BMG [27]. In this study, we selected Cu particles with a relatively higher thermal conductivity as a spacer to prevent crystallization in porous amorphous materials caused by a longer holding time and a higher hot press temperature. As shown in Figure 2, the matrix of Ti_42_Zr_35_Ta_3_Si_5_Co_12.5_Sn_2.5_ BMG foams with different porosities did not exhibit a crystalline peak but typically broad diffraction peaks that represented the amorphous state of the matrix like the other amorphous materials [28,29]. Figure 1 shows that additional Cu particles increased thermal conductivity, and the hot press temperature, holding time, and pressure could be decreased based on the same bonding interface.

The mechanical properties of metallic materials used in the orthopedic field are usually substantially high and may cause a stress-shielding effect. The mechanical properties of fully dense materials can be modified through chemical composition adjustment, microstructure changes, and surface treatment. However, the porosity of porous materials and the degree of junction between particles can be controlled to adjust mechanical properties [30]. 

Our results demonstrated that the compressive strength of Ti_42_Zr_35_Ta_3_Si_5_Co_12.5_Sn_2.5_ BMG foams decreased with an increase in the volume fraction of Cu spacer particles (Figure 6). The findings indicated that the mechanical properties of foam material can be controlled by adjusting the porosity and the degree of junction between particles.

Fabrication of porous materials can be an excellent strategy for achieving biological fixation and improving orthopedic implants’ longevity [5]. An ideal porous orthopedic implant should have macro (>100 µm)- and micro (<20 µm)-sized pores, which must be interconnected. The multidimensional porosity of implants was more favorable than only the one-dimensional porosity of implants. Woodard et al. compared the relative osteoconductivity and changes in the mechanical properties of hydroxyapatite scaffolds with multiscale porosity with those of scaffolds with a single pore size. They found that only the implant with multiscale porosity contained the bone. Moreover, the strength and stiffness of the implant with a single pore size decreased by 15% and 46%, respectively, and those of the implant with multiscale porosity decreased by 30% and 31%, respectively [8]. This crucial finding described the relationship not only between multiple pore sizes and osteoconductivity but also between multiple pore sizes and mechanical properties. Moreover, the micro-sized pores can provide an effective drug delivery mechanism. According to the above report, using a nonuniform size of spacer particles to fabricate the Ti_42_Zr_35_Ta_3_Si_5_Co_12.5_Sn_2.5_ BMG foam will be interesting and attractive in the future.

In this study, the in vitro biocompatibility and biological response of different porosities of Ti_42_Zr_35_Ta_3_Si_5_Co_12.5_Sn_2.5_ BMG foams were found to have no significant difference (Figure 7, Figure 8 and Figure 9) because the difference between different groups was only the porosity, not the composition of materials. In some biomedical applications, porous materials are usually used as drug delivery materials [31,32]. Recently, many different metallic materials have been used to develop and manufacture porous scaffolds, including biodegradable and non-biodegradable metals, such as Fe alloy, Mg alloy, Zn alloy, 316 L stainless steel, Ti alloy, Co-Cr alloy, and Ta alloy [33,34,35,36,37,38,39]. In clinical applications, Ti-based alloys are the most popular and widely used in orthopedics or the dental field because of their excellent biocompatibility. Moreover, Xue et al. have reported that the level of alkaline phosphatase expression on a porous Ti scaffold was much higher than that on a Ti sheet [40], which means that the porous scaffold can be a benefit for faster integration with the bone tissue [40].

## 5. Conclusions

In the present study, we fabricated porous Ti_42_Zr_35_Ta_3_Si_5_Co_12.5_Sn_2.5_ BMG foams with Cu spacer particles by using the hot press method. The increase in thermal conductivity could shorten the holding time in the fabrication process. Moreover, the compressive strength could be adjusted by controlling the porosity, and it was predictable. TiZr-based BMG foams demonstrated favorable biocompatibility and have a great potential for orthopedic implant applications.

## Figures and Tables

**Table 1:** Real porosity of Ti_42_Zr_35_Ta_3_Si_5_Co_12.5_Sn_2.5_ bulk metallic glass foams fabricated using different volume fractions of Cu spacer particles.

Volume Fraction of Cu (vol.%)	0	10	20	30	40	50
Real Porosity (%)	2.0	11.1	27.6	40.4	51.0	67.9

**Table 2:** Thermal conductivity of Ti_42_Zr_35_Ta_3_Si_5_Co_12.5_Sn_2.5_ bulk metallic glass foams fabricated using different volume fractions of Cu spacer particles (unit: W/m°C).

	Volume Fraction of Cu Particles	10%	20%	30%	40%	50%
Temperature (°C)						
50		10.78	21.62	29.54	35.68	52.29
100		12.96	25.43	34.65	48.12	58.87
150		13.93	26.37	36.52	52.99	65.19
200		15.03	27.56	38.85	58.70	69.28
250		16.49	29.04	41.43	64.83	73.44
275		17.23	29.78	42.79	67.83	75.49
300		17.73	30.32	43.99	68.01	75.33
325		18.04	30.18	44.47	68.61	75.38
350		18.64	29.45	45.13	69.48	75.84
375		19.26	29.15	45.39	70.52	76.11
400		19.94	28.38	45.59	69.05	76.63
425		20.58	27.74	45.78	70.19	75.41
450		19.89	25.90	44.28	69.05	73.57

**Table 3:** Glass-forming abilities of Ti_42_Zr_35_Ta_3_Si_5_Co_12.5_Sn_2.5_ bulk metallic glass foams with different porosities fabricated using Cu spacer particles at a heating rate of 40 K/min.

Volume Fraction of Cu (vol.%)	$T_{g}\;(K)$	$T_{x}\;(K)$	$\Delta T_{x}\;(K)$
0	726	832	106
10	743	837	94
20	742	838	96
30	744	839	95
40	740	839	99
50	741	840	99

**Table 4:** Mechanical properties of Ti_42_Zr_35_Ta_3_Si_5_Co_12.5_Sn_2.5_ bulk metallic glass foams with different porosities fabricated using different volume fractions of Cu spacer particles.

Volume Fraction of Cu (vol.%)	E (GPa)	σ (GPa)	$\frac{E}{E_{s}}$	$\frac{\sigma }{\sigma_{s}}$	$\frac{\rho }{\rho_{s}}$	${\left(\frac{\rho }{\rho_{s}}\right)}^{3.5}$	${\left(\frac{\rho }{\rho_{s}}\right)}^{4}$
0	79.7	1261	0.71	0.94	0.98	0.93	0.92
10	44.5	679	0.40	0.51	0.84	0.53	0.49
20	21.4	388	0.19	0.29	0.69	0.28	0.23
30	11.2	214	0.10	0.16	0.60	0.17	0.13
40	9.4	143	0.08	0.11	0.46	0.06	0.04
50	4.6	76	0.04	0.06	0.30	0.02	0.009

## Data Availability

The data presented in this study are available on request from the corresponding authors. The data are not publicly available since they are raw data.

## References

[1] Goodman S.B., Yao Z., Keeney M., Yang F. (2013). The future of biologic coatings for orthopaedic implants. Biomaterials.

[2] Borcherding K., Schmidmaier G., Hofmann G.O., Wildemann B. (2020). The rationale behind implant coatings to promote osteointegration, bone healing or regeneration. Injury.

[3] Yeo I.-S.L. (2019). Modifications of Dental Implant Surfaces at the Micro- and Nano-Level for Enhanced Osseointegration. Materials.

[4] Kienapfel H., Sprey C., Wilke A., Griss P. (1999). Implant fixation by bone ingrowth. J. Arthroplast..

[5] Matassi F., Botti A., Sirleo L., Carulli C., Innocenti M. (2013). Porous metal for orthopedics implants. Clin. Cases Miner. Bone Metab..

[6] Bose S., Roy M., Bandyopadhyay A. (2012). Recent advances in bone tissue engineering scaffolds. Trends Biotechnol..

[7] Matassi F., Nistri L., Paez D.C., Innocenti M. (2011). New biomaterials for bone regeneration. Clin. Cases Miner. Bone Metab..

[8] Woodard J.R., Hilldore A.J., Lan S.K., Park C., Morgan A.W., Eurell J.A.C., Clark S.G., Wheeler M., Jamison R.D., Johnson A.J.W. (2007). The mechanical properties and osteoconductivity of hydroxyapatite bone scaffolds with multi-scale porosity. Biomaterials.

[9] Nouri A., Hodgson P.D., Wen C. (2010). Biomimetic Porous Titanium Scaffolds for Orthopedic and Dental Applications. Biomimetics Learning from Nature.

[10] Akahori T., Niinomi M., Nakai M., Fukuda H., Fukui H., Ogawa M. (2007). Bioactive Ceramic Surface Modification of β-Type Ti-Nb-Ta-Zr System Alloy by Alkali Solution Treatment. Mater. Trans..

[11] Gepreel M.A.-H., Niinomi M. (2013). Biocompatibility of Ti-alloys for long-term implantation. J. Mech. Behav. Biomed. Mater..

[12] Li T., Wong P., Chang S., Tsai P., Jang J., Huang J. (2017). Biocompatibility study on Ni-free Ti-based and Zr-based bulk metallic glasses. Mater. Sci. Eng. C.

[13] Nguyen V.T., Wong X.P.-C., Song S.-M., Tsai P.-H., Jang J.S.-C., Tsao I.-Y., Lin C.-H., Nguyen V.C. (2020). Open-Cell Tizr-Based Bulk Metallic Glass Scaffolds with Excellent Biocompatibility and Suitable Me-chanical Properties for Biomedical Application. J. Funct. Biomater..

[14] Nguyen V.T., Li T.H., Song S.M., Liao Y.C., Tsai P.H., Wong P.C., Nguyen V.C., Jang J.S.C. (2019). Synthesis of biocompatible TiZr-based bulk metallic glass foams for bio-implant application. Mater. Lett..

[15] Kujala S., Ryhänen J., Danilov A., Tuukkanen J. (2003). Effect of porosity on the osteointegration and bone ingrowth of a weight-bearing nickel–titanium bone graft substitute. Biomaterials.

[16] Swieczko-Zurek B. (2009). Porous Materials Used as Inserted Bone Implants. Adv. Mater. Sci..

[17] Ma G.-F., Ali A., Verzijl N., Hanemaaijer R., Tekoppele J., Konttinen Y.T., Salo J. (2006). Increased collagen degradation around loosened total hip replacement implants. Arthritis Care Res..

[18] Lv Y., Wang B., Liu G., Tang Y., Lu E., Xie K., Lan C., Liu J., Qin Z., Wang L. (2021). Metal Material, Properties and Design Methods of Porous Biomedical Scaffolds for Additive Manufacturing: A Review. Front. Bioeng. Biotechnol..

[19] Chen Z., Yan X., Yin S., Liu L., Liu X., Zhao G., Ma W., Qi W., Ren Z., Liao H. (2020). Influence of the pore size and porosity of selective laser melted Ti6Al4V ELI porous scaffold on cell proliferation, osteogenesis and bone ingrowth. Mater. Sci. Eng. C.

[20] Szymczyk-Ziółkowska P., Łabowska M.B., Detyna J., Michalak I., Gruber P. (2020). A review of fabrication polymer scaffolds for biomedical applications using additive manufacturing techniques. Biocybern. Biomed. Eng..

[21] Bose S., Ke D., Sahasrabudhe H., Bandyopadhyay A. (2017). Additive manufacturing of biomaterials. Prog. Mater. Sci..

[22] Li X. (2017). Additive Manufacturing of Advanced Multi-Component Alloys: Bulk Metallic Glasses and High Entropy Alloys. Adv. Eng. Mater..

[23] Zhang P., Tan J., Tian Y., Yan H., Yu Z. (2021). Research progress on selective laser melting (SLM) of bulk metallic glasses (BMGs): A review. Int. J. Adv. Manuf. Technol..

[24] Wang H.-S., Li T.-H., Chen H.-G., Pan J.-H., Jang J.S.-C. (2019). Microstructural evolution and properties of laser spot-welded Zr Al Co Ta bulk metallic glass under various initial welding temperatures. Intermetallics.

[25] (1999). Biological Evaluation of Medical Devices Part 5: Test for Cytotoxicity: In Vitro Methods.

[26] Kiran G., Suman K., Rao N., Rao R. (2011). A study on the influence of hot press forming process parameters on mechanical properties of green composites using Taguchi experimental design. Int. J. Eng. Sci. Technol..

[27] Abreu A.C., Tavares R.R., Borges A., Mergulhão F., Simões M. (2013). Current and emergent strategies for disinfection of hospital environments. J. Antimicrob. Chemother..

[28] Song S.-M., Wong P.-C., Chiang C.-W., Tsai P.-H., Jang J.S.C., Chen C.-H. (2020). A bi-phase core-shell structure of Mg-based bulk metallic glass for application in orthopedic fixation im-plants. Mater Sci. Eng. C Mater. Biol. Appl..

[29] Wong P.-C., Tsai P.-H., Li T.-H., Cheng C.-K., Jang J., Huang J. (2017). Degradation behavior and mechanical strength of Mg-Zn-Ca bulk metallic glass composites with Ti particles as biodegradable materials. J. Alloys Compd..

[30] Ternero F., Rosa L.G., Urban P., Montes J.M., Cuevas F.G. (2021). Influence of the Total Porosity on the Properties of Sintered Materials—A Review. Metals.

[31] Unamuno X., Imbuluzqueta E., Salles F., Horcajada P., Blanco-Prieto M. (2018). Biocompatible porous metal-organic framework nanoparticles based on Fe or Zr for gentamicin vectorization. Eur. J. Pharm. Biopharm..

[32] Matsuyama K., Hayashi N., Yokomizo M., Kato T., Ohara K., Okuyama T. (2014). Supercritical carbon dioxide-assisted drug loading and release from biocompatible porous metal–organic frameworks. J. Mater. Chem. B.

[33] Li Y.J.H., Zhou J., Zadpoor A.A. (2020). Additively manufactured biodegradable porous metals. Acta Biomater..

[34] Ma S., Tang Q., Feng Q., Song J., Han X., Guo F. (2019). Mechanical behaviours and mass transport properties of bone-mimicking scaffolds consisted of gyroid structures manufactured using selective laser melting. J. Mech. Behav. Biomed. Mater..

[35] Yamamoto A., Kohyama Y., Kuroda D., Hanawa T. (2004). Cytocompatibility evaluation of Ni-free stainless steel manufactured by nitrogen adsorption treatment. Mater. Sci. Eng. C.

[36] Cutolo A., Engelen B., Desmet W., Van Hooreweder B. (2020). Mechanical properties of diamond lattice Ti–6Al–4V structures produced by laser powder bed fusion: On the effect of the load direction. J. Mech. Behav. Biomed. Mater..

[37] Wauthle R., van der Stok J., Yavari S.A., Van Humbeeck J., Kruth J.-P., Zadpoor A.A., Weinans H., Mulier M., Schrooten J. (2015). Additively manufactured porous tantalum implants. Acta Biomater..

[38] Yan C., Hao L., Hussein A., Young P., Raymont D. (2014). Advanced lightweight 316L stainless steel cellular lattice structures fabricated via selective laser melting. Mater. Des..

[39] Demir A.G., Previtali B. (2017). Additive manufacturing of cardiovascular CoCr stents by selective laser melting. Mater. Des..

[40] Xue W., Krishna B.V., Bandyopadhyay A., Bose S. (2007). Processing and biocompatibility evaluation of laser processed porous titanium. Acta Biomater..

